# Learning Lessons From Long-Term Care Policy Financing Efforts in the United States

**DOI:** 10.1093/geroni/igaa057.2366

**Published:** 2020-12-16

**Authors:** Michael Lepore

**Affiliations:** LiveWell Alliance, Southington, Connecticut, United States

## Abstract

A decades-long policy impasse has crippled our national capacity to finance long-term care (LTC) sufficiently or equitably, leaving large swaths of the US population at risk of going broke paying privately for LTC or having unmet LTC needs, while also draining state and federal budgets. By reviewing past LTC financing policy efforts—from the passage of the Social Security Act and the enactment of Medicaid and Medicare, to the LTC financing proposals advanced by 2020 presidential candidates—the political interplay of budgetary concerns in government spending and social justice concerns regarding access to care emerged as a primary LTC policymaking issue. Establishing national consensus on the prioritization of these fiscal and social justice concerns, and their respective values, could help lawmakers craft policy capable of generating the political will needed to overcome political gridlock. Clarifying how LTC benefits would be paid for appears to be a relatively straightforward technical task in comparison.

